# A Decade-Long Retrospective Clinicopathological Study of Appendiceal Neoplasms

**DOI:** 10.7759/cureus.70778

**Published:** 2024-10-03

**Authors:** Priyadharshini R, Shobini Vishali V M, Sulochana Sonti

**Affiliations:** 1 Department of Pathology, Saveetha Medical College and Hospital, Saveetha Institute of Medical and Technical Sciences, Saveetha University, Chennai, IND

**Keywords:** appendiceal adenocarcinoma, appendiceal neuroendocrine tumor, carcinoma of the appendix, low-grade appendiceal mucinous neoplasm, non-hodgkin's lymphoma

## Abstract

Introduction: Appendiceal neoplasms (ANs) are uncommon, representing less than 1% of all gastrointestinal tumors. They are most often discovered incidentally during appendectomies performed for suspected acute appendicitis. Recent studies have reported an increase in the incidence of AN. Our study aimed to analyze the spectrum of AN and determine the cases discovered incidentally.

Material and methods: This retrospective descriptive study was performed over 10 years, from March 2014 to March 2024, at Saveetha Medical College and Hospital in Chennai, India. Complete enumeration sampling was performed, and tumor registries were reviewed to identify all cases of AN received during the study period. The corresponding clinical and radiological data were obtained from the hospital database, whereas histopathology records were used to evaluate tumor characteristics. Descriptive statistical analysis was employed, and the spectrum of AN was analyzed.

Results: During the study period, 12 cases of AN were identified. Eleven cases (91.7%) were diagnosed from appendicectomy specimens, while one was from a right hemicolectomy specimen. Of these 12, nine cases (75%) were incidental findings. In our study, we found that there was a higher number of female cases, with nine out of twelve cases (75%) being female. Most patients presented with abdominal pain, and acute appendicitis was the most frequent preoperative diagnosis. Low-grade appendiceal mucinous neoplasm was the most common type encountered followed by other appendiceal tumors.

Conclusion: The incidence of AN has increased in recent years, with most cases being found incidentally. Given the increasing rate of incidental AN, it is vital to maintain vigilance in specimen analysis including comprehensive examination and precise grossing to ensure malignancies are not overlooked.

## Introduction

Neoplasms involving the appendix are rare and represent less than 1% of gastrointestinal tumors (approximately between 0.2% and 0.5%) [1,2]. It is identified in approximately 1%-2% of appendicectomy specimens performed in cases of suspected appendicitis [3]. Appendiceal neoplasms (ANs) encompass many pathologies, including epithelial and nonepithelial tumors [4]. According to the fifth edition of the WHO Classification of Tumors in the gastrointestinal tract (GIT), AN can be broadly categorized into serrated lesions and polyps, mucinous neoplasms, adenocarcinoma, goblet cell adenocarcinoma, and neuroendocrine neoplasms (NENs) [5]. In addition, the lymphoid tissue within the mucosal and submucosal layers of the appendix can lead to the formation of lymphomas [6].

Despite the wide spectrum, AN often presents with symptoms like appendicitis. The neoplasms are typically identified postoperatively through histopathological examination of the excised appendix, as preoperative and intraoperative diagnoses are rarely made [1,7-9]. Although abdominal ultrasonography and computed tomography aid in diagnosis, detecting these conditions before surgery is rare [10]. Diagnosis can be particularly challenging in women, as primary appendiceal and ovarian mucinous neoplasms often exhibit similar atypical clinical and imaging findings [11]. The rarity of their occurrence and subtlety of symptoms present challenges, often causing delays in diagnosis and making treatment decisions more difficult. Recent reports highlight a reduction in the submission of appendicectomy specimens for histopathological analysis and a growing preference for nonoperative management of acute appendicitis [12,13]. These practices can lead to undiagnosed tumors, even after surgical intervention. The decline in tissue submission for examination has coincided with an increase in studies documenting recurrent neoplasms in the appendiceal remnant [12]. Thus, histopathological evaluation plays a crucial role in diagnosing AN.

The rarity of appendiceal tumors has led to less extensive research compared to other gastrointestinal tumors. Despite this, their incidence has been steadily increasing over the past decade [14]. The tumors show variation in the presentation; appendiceal neuroendocrine tumors are typically found in people younger than 50 years, while other subtypes of appendiceal cancer tend to occur more often in older individuals [14]. In the past, neuroendocrine tumors were the primary type of appendiceal tumors. However, trends have changed, with epithelial neoplasms now being more prevalent [2]. This study aims to explore the histopathological spectrum of AN with age, gender, clinical presentation, and radiologic findings and to determine the cases of AN discovered incidentally.

## Materials and methods

This study was conducted in the Department of Pathology, Saveetha Medical College and Hospital, Chennai. It is a retrospective descriptive study performed over 10 years between March 2014 and March 2024. A complete enumeration sampling technique was employed. All cases of AN were included in the study, regardless of the patient's age, gender, or the type of surgical intervention the patient underwent. Our study also included those cases where AN was diagnosed concurrently with other malignancies. Cases with incomplete or unavailable data on clinical presentation and imaging findings were excluded. Additionally, those cases lacking slides or tissue blocks in our department's archives were also excluded. Tumor registers from the corresponding years were meticulously reviewed, and 12 cases of AN were identified based on the established inclusion and exclusion criteria. Demographic, clinical, and radiological details, along with histopathological findings, were reviewed for all the cases under the study.

Demographic details were obtained from the requisition form sent along with the specimens. The specimens were received in 10% neutral buffered formalin. Following overnight fixation, the specimens were grossed according to the standard protocol. The tissue samples were then processed through dehydration, clearing, and embedding in paraffin, forming a solid suitable for sectioning. Thin sections of the paraffin-embedded tissue, measuring 4-5 µm in thickness, were cut using a microtome, and slides were stained according to standard techniques. The routine histopathological analysis included examination of hematoxylin and eosin (H&E)-stained slides. Immunohistochemistry was performed in cases where the initial evaluation on H&E suggested the need for further analysis. Histopathological findings and final diagnosis were retrieved from the histopathology records maintained by our department. The corresponding clinical data, including presenting complaints, preoperative impressions, radiological findings, and surgery performed, were accessed from the hospital database using the patient’s unique identity number.

The collected data, which included age, gender, presenting complaints, preoperative diagnosis, radiology findings, surgery performed, and histopathological diagnosis, were recorded in a Microsoft Excel spreadsheet. Descriptive statistical analysis was employed, with continuous variables represented using the mean, median, and standard deviation, and categorical variables presented as frequencies and percentages. The spectrum of AN with respect to age, gender, clinical presentation, and radiologic findings was analyzed, and the cases of AN discovered incidentally were determined.

## Results

**Figure 1:**
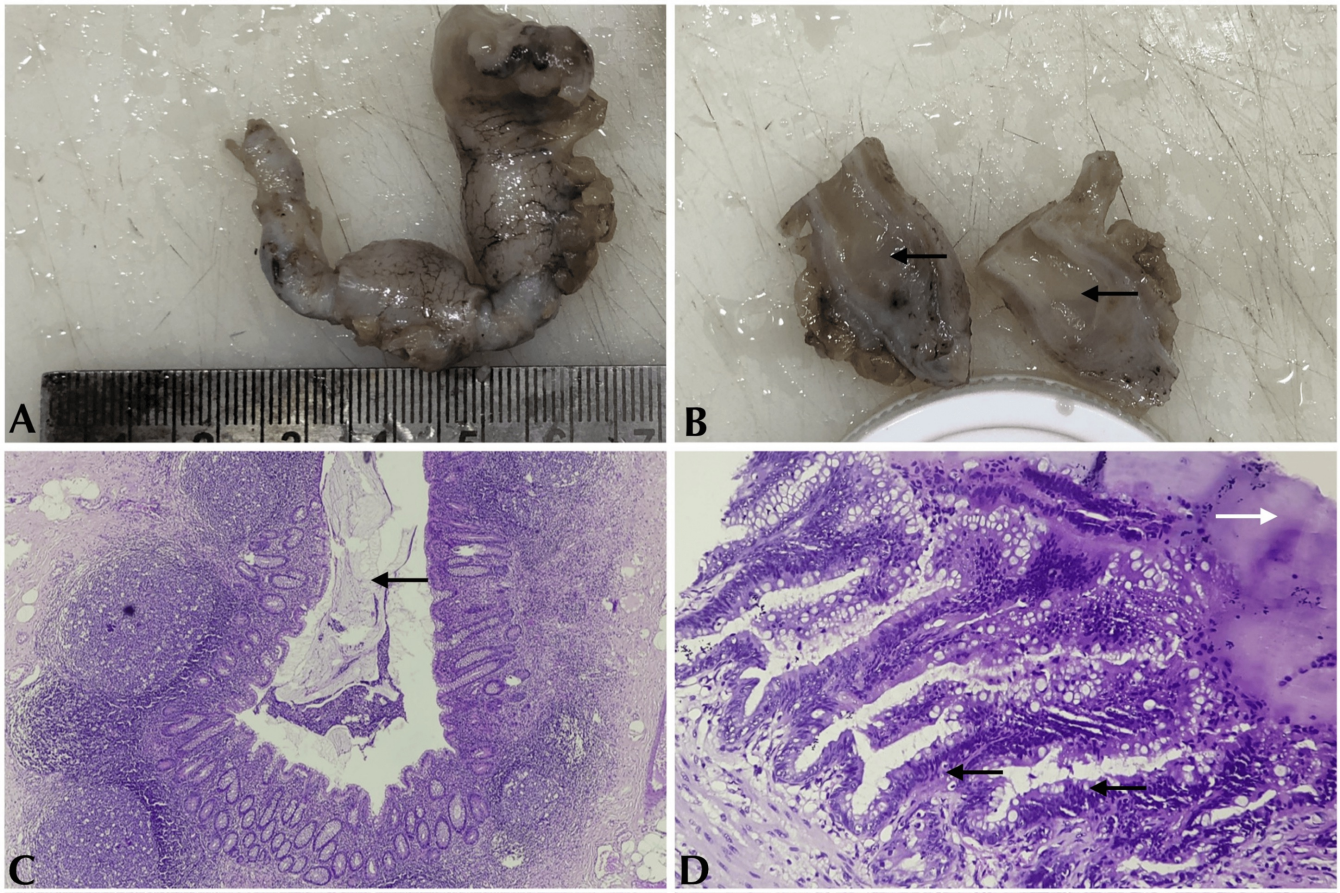
LAMN. (A) Gross appearance. (B) Cut section: lumen appears dilated and filled with mucinous material (black arrows). (C) Appendiceal lumen showing mucinous material (4x magnification, H&E) (black arrows). (D) Appendiceal mucosa showing mucin (white arrow) and nuclear stratification (black arrows) (40x magnification, H&E) (black arrows) LAMN: low-grade appendiceal mucinous neoplasms; H&E: hematoxylin and eosin

During the study period, a total of 12 cases of AN were documented. Low-grade appendiceal mucinous neoplasm (LAMN) constituted seven cases (58.4%), followed by neuroendocrine tumors at three cases (25%), and both appendiceal adenocarcinoma and non-Hodgkin's lymphoma each comprised one case (8.3%). The most commonly encountered AN, LAMN, is represented in Figure 1.

Clinical presentation and radiological finding

Most patients presented to the emergency department with abdominal pain as their primary complaint. The most common preoperative diagnosis was acute appendicitis, followed by appendiceal lesions. Of the 12 cases, three (25%) showed an appendiceal lesion on radiological imaging. Table 1 provides an overall summary of ANs.

**Table 1:** Overview of ANs RIF: right iliac fossa; USG: ultrasound; CT: computed tomography; NET: neuroendocrine tumor; LAMN: low-grade appendiceal mucinous neoplasms; CECT: contrast-enhanced computed tomography; ANs: appendiceal neoplasm

Patient	Age/gender	Presentation	Preoperative diagnosis	Imaging	Surgery performed	Pathological impression
1	45/M	RIF pain	Acute appendicitis	USG and CT: acute appendicitis with periappendicular abscess	Appendicectomy	Adenocarcinoma grade 2 (pT4apNx)
2	67/F	Abdominal pain and nausea	Torsion of the right ovarian mass	CT: benign serous cystadenoma with features of torsion	Emergency staging laparotomy with appendicectomy	Well-differentiated NET and right benign serous cystadenoma of the ovary
3	46/F	RIF pain	Acute appendicitis	USG: acute appendicitis	Appendicectomy	Well-differentiated NET
4	25/F	RIF pain	Acute appendicitis	USG: acute appendicitis	Appendicectomy	Well-differentiated NET
5	47/F	RIF pain	Acute appendicitis	USG: acute appendicitis	Appendicectomy	LAMN
6	61/F	RIF pain	Acute appendicitis	USG: acute appendicitis	Appendicectomy	LAMN
7	39/M	RIF pain	Acute appendicitis	CT: appendiceal mucocele	Appendicectomy	LAMN
8	21/F	RIF pain, fever, and nausea	Acute appendicitis	USG: appendiceal mucocele	Appendicectomy	LAMN
9	55/F	Abdominal distension and pain	Right ovarian cyst	CECT: right ovarian malignancy	Laparotomy with total abdominal hysterectomy with bilateral salpingo-oophorectomy with appendicectomy	LAMN and Brenner tumor of bilateral ovaries
10	77/F	Abdominal pain	Acute appendicitis	USG: acute appendicitis	Appendicectomy	LAMN
11	22/M	RIF pain	Acute appendicitis	USG: acute appendicitis	Appendicectomy	LAMN
12	78/F	Abdominal pain and blood in stools	Appendicular mass	CECT: features of appendicular malignancy	Right hemicolectomy with ileocolic anastomosis	Non-Hodgkin's lymphoma

Age and gender

**Figure 2:**
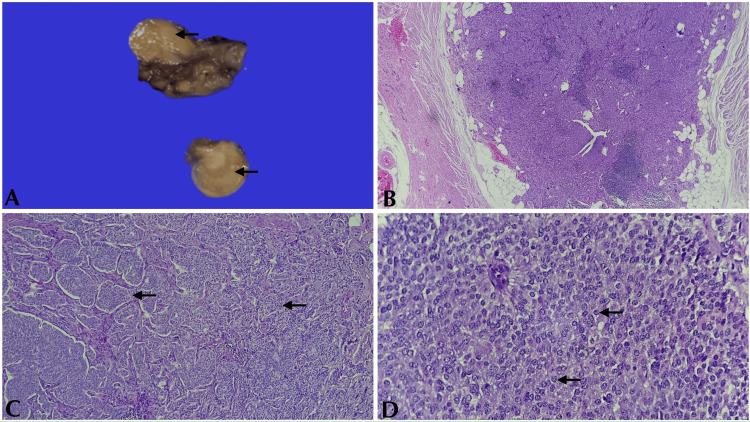
NET. (A) Cut surface of the appendix showing a yellow lesion (black arrows). (B) NET (4x magnification, H&E). (C) Nests of tumor cells (20x magnification, H&E) (black arrows). (D) Cells showing finely stippled chromatin (40x magnification, H&E) (black arrows) NET: neuroendocrine tumor; H&E: hematoxylin and eosin

The age at diagnosis spanned from 21 to 78 years, with a median age of 48.5 years. Of these cases, three (25%) were observed in men and nine (75%) in women with a ratio of 1:3. LAMN had a female predilection (71.4%) with an average age of presentation of 46 ± 20.5 years. Appendiceal neuroendocrine tumors were exclusively observed in females with an average age of presentation of 46 ± 21 years. A 45-year-old male was diagnosed with adenocarcinoma, while a 78-year-old female was diagnosed with lymphoma. Figure 2 depicts a case of appendiceal neuroendocrine tumor.

Surgical procedure

**Figure 3:**
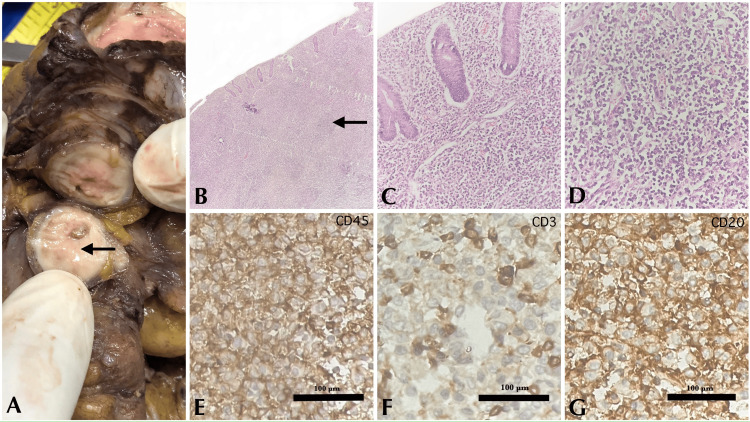
NHL. (A) Gross appearance showing a gray-white lesion (black arrows). (B) NHL showing appendiceal mucosa with lymphoid follicles (4x magnification, H&E) (black arrows). (C) Sheets of lymphoid cells (20x magnification, H&E). (D) Lymphoid cells with scant cytoplasm and irregular nuclear membrane (40x magnification, H&E). (E) CD45 showing diffuse positivity (40x magnification, IHC). (F) CD3 showing scattered positivity in reactive lymphocytes (40x magnification, IHC). (G) CD20 showing diffuse positivity (40x magnification, IHC) NHL: Non-Hodgkin's lymphoma; H&E: hematoxylin and eosin; IHC: immunohistochemistry

Among the 12 cases of appendiceal tumors, 11 cases (91.7%) were identified in appendicectomy specimens, and one case (8.3%) was observed in a right hemicolectomy specimen performed for a radiologically confirmed appendicular lesion. Among these 11 tumors identified on appendicectomy specimens, seven cases (63.6%) were found in appendectomies performed for suspected appendicitis, two cases (18.2%) for suspected appendiceal mucoceles, and two cases (18.2%) were discovered in prophylactic appendectomies performed for ovarian malignancies suspected to be mucinous neoplasms. Figure 3 represents a case of non-Hodgkin's lymphoma involving the appendix.

Incidental ANs

**Figure 4:**
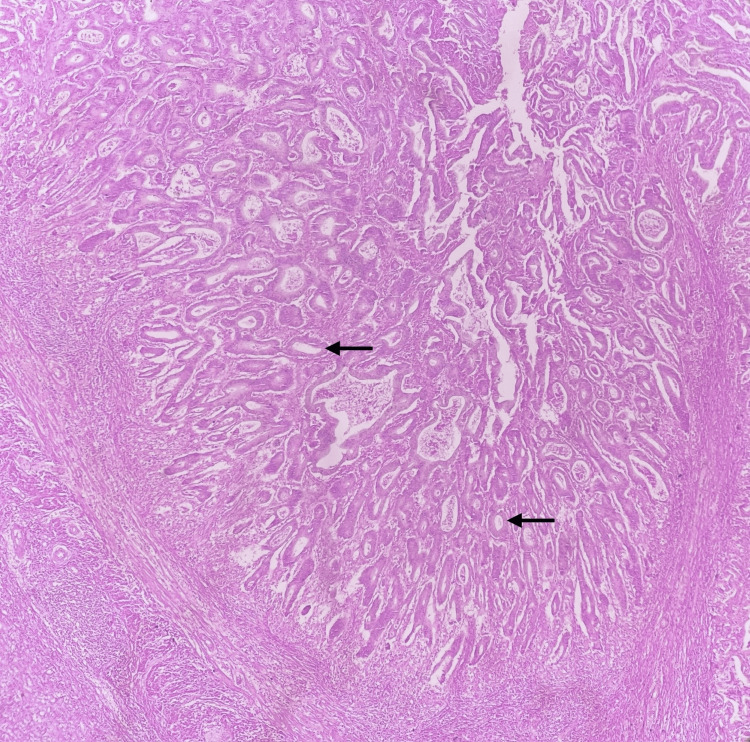
Appendiceal adenocarcinoma showing glands lined by pleomorphic cells. Arrows highlight the glands (20x magnification, H&E) H&E: hematoxylin and eosin

Nine specimens (0.32%) were incidentally diagnosed as AN. Of these neoplasms, LAMN constituted five cases (55.6%), neuroendocrine tumors accounted for three cases (33.3%), and adenocarcinoma was found in one case (11.1%). Figure 4 displays a case of appendiceal adenocarcinoma.

## Discussion

The median age of presentation was 48.5 years, comparable to the 49 years reported by Skendelas et al. [13], contrasting with the median age of 63 years observed by Gómez-Báez et al. [2]. Previous research has identified factors like female gender to be associated with malignancy. Similarly, our study also found a higher incidence of appendiceal tumors in females, consistent with the findings by Rencuzogullari et al. [15] and Yilmaz and Bolukbasi [16], in contrast to the findings of Kunduz et al. [17], who observed a higher incidence of AN in men. Few studies indicate a higher incidence of AN in recent times [3,9,12]. While the overall incidence of AN in our study is lower than previously reported figures, we observed an increase in cases over the last five years, with nine cases identified.

Consistent with findings from other studies, preoperative diagnosis of AN was infrequently achieved in our series of patients [3,6]. In only 25% of the cases (three out of 12), preoperative imaging either favored or suggested the possibility of neoplasms. One of these cases was non-Hodgkin's lymphoma, whereas the other two presented with LAMN morphology. Abdominal pain was the chief complaint among the majority of patients presenting to the emergency department, concordant with the observations of Gómez-Báez et al. [2], Boyajian et al. [6], and Tajima et al. [12]. Only a small subset (two out of 12 cases) exhibited symptoms of fever and nausea in association with abdominal pain. Acute appendicitis was the most frequent preoperative diagnosis (58.4%), consistent with findings by Arellano et al. [18] and Chen et al. [19].

Mucinous neoplasms in the appendix are of low and high grades. LAMN is uncommon, with an incidence of 0.7%-1.7% in all appendicectomy specimens [11]. Our study showed an increased incidence of LAMN among other ANs, a finding concordant with a study by Gómez-Báez et al. [2]. Among the seven cases of LAMN, five were incidental findings. About 50% of the LAMN cases are diagnosed in asymptomatic patients, particularly among women undergoing appendectomy during gynecological procedures [20]. Likewise, one among the seven cases of LAMN was incidentally found in a prophylactic appendicectomy done in a woman with bilateral ovarian malignancy (Brenner tumor). While LAMN usually has a favorable prognosis, it can progress to pseudomyxoma peritonei if not detected, leading to potential complications [20].

NENs most commonly develop in the small intestine and rectum, with the appendix being the third most frequent site in the GIT. These neoplasms originate from subepithelial neuroendocrine cells, particularly concentrated at the tip of the appendix, which explains their predilection for this location [4]. The incidence of appendiceal NENs in appendicectomy specimens is 0.30%-2.27%. Our study showed an incidence of 0.10%. As with the study by Barut and Gönültaş, none of our patients were suspected of having NENs preoperatively [21]. Appendiceal carcinoid tumors are typically diagnosed more frequently in females than in males. Consistent with these findings, our study also demonstrated exclusive involvement of NEN in women. However, a study by Tchana-Sato et al. and Zhang et al. reported a male predominance, which contrasts with our results [22,23]. All three NEN cases in our study were incidental findings: two were detected during appendectomies for suspected appendicitis, and one was in a prophylactic appendicectomy in a patient with a right ovarian malignancy (serous cystadenoma with torsion).

A case of appendiceal adenocarcinoma and a non-Hodgkin's lymphoma were also reported. Primary adenocarcinoma of the appendix occurs infrequently, representing only 0.08%-0.2% of all appendicectomy specimens [7]. Adenocarcinoma was not preoperatively diagnosed and presented as acute appendicitis, while the case of lymphoma was detected to be a malignancy during radiological imaging. Primary adenocarcinoma of the appendix is commonly associated with a risk of perforation [18]. Approximately 40% of these appendiceal adenocarcinoma cases are diagnosed during emergency appendectomies that are initially performed for suspected acute appendicitis or a periappendiceal abscess [7]. Lymphomas involving the small and large intestines are uncommon [24]. Primary lymphoma of the appendix is a particularly rare condition [25], representing just 0.015% of GIT lymphomas [26]. Though rare, a case of lymphoma can also be suspected in an individual who presents with an appendicular lesion.

The majority of the cases of AN in our study were incidental findings (75%). The newer trends in the management of acute appendicitis have led to concerns about the potential risk of missing ANs in patients treated nonsurgically [27,28]. Though appendicectomy stands as a gold standard for confirming appendiceal cancer, there are no standardized protocols for grossing appendectomy specimens, which presents a unique challenge in diagnosing malignancy in cases suspected of appendicitis [3]. At our institution, we take three representative sections of the appendix, though this practice varies globally. The three sections include a longitudinal section of the tip of the appendix and transverse sections from the surgically resected end and the center of the appendix. A study by Bahmad et al. suggests that taking five representative sections can increase the likelihood of diagnosing neoplasms in cases of acute appendicitis where malignancy was not initially suspected [3]. Therefore, in cases of suspected appendicitis, concurrent ovarian malignancies, and lesions of the right colon, a thorough examination and meticulous grossing of specimens are crucial to rule out ANs.

Our study faced certain limitations. To begin with, it is a retrospective analysis from a single center. Although we captured all cases of surgically treated AN at our facility, the sample size was limited, reflecting the disease's low incidence. Additionally, we did not perform long-term follow-ups on our AN patients to determine their outcomes or on those who received medical treatment, which may have yielded more comprehensive insights into the risks of overlooked AN cases.

## Conclusions

AN though relatively rare has demonstrated an increasing trend in our study over the recent years, with the majority of cases found in females. Preoperative diagnosis of AN was rarely observed with most neoplasms being incidentally discovered during appendectomies initially performed for suspected acute appendicitis, where abdominal pain was a frequent symptom. The most common neoplasm observed was LAMN, followed by neuroendocrine tumors. Diagnosing appendiceal tumors before surgery poses a considerable challenge, particularly in situations where ovarian malignancy is also present. The clinical overlap in presentation between these two conditions often complicates preoperative diagnosis, making it difficult to distinguish between them without surgical intervention. Given these challenges, a thorough examination and meticulous grossing of specimens are crucial in cases of suspected appendicitis, concurrent ovarian malignancies, and lesions of the right colon.

## References

[1] Issin G, Demir F, Guvendir Bakkaloglu I, Cagatay DV, Aktug Simsek H, Yilmaz I, Zemheri E (2023). High incidence of appendiceal neoplasms in the elderly: a critical concern for non-surgical treatment. Med Princ Pract.

[2] Gómez-Báez FD, Cerdán-Santacruz C, Muguiro NM (2023). Incidence, clinicopathological features and oncologic outcome of appendiceal neoplasms: a single-center cohort study. Gastrointest Disord.

[3] Bahmad HF, Aljamal AA, Alvarez Moreno JC, Salami A, Bao P, Alghamdi S, Poppiti RJ (2021). Rising incidence of appendiceal neoplasms over time: does pathological handling of appendectomy specimens play a role?. Ann Diagn Pathol.

[4] Rossi A, Maloney Patel N (2023). Appendiceal neoplasms-a practical guide. J Surg Oncol.

[5] Ahadi M, Sokolova A, Brown I, Chou A, Gill AJ (2021). The 2019 World Health Organization Classification of appendiceal, colorectal and anal canal tumours: an update and critical assessment. Pathology.

[6] Boyajian H, Majeski V, Flores A, Sturtz D, Baidoun F, Dughayli M (2020). Clinicopathological and perioperative outcome of appendiceal tumors: case review of 31 patients. Spartan Med Res J.

[7] Lee WS, Choi ST, Lee JN, Kim KK, Park YH, Baek JH (2011). A retrospective clinicopathological analysis of appendiceal tumors from 3,744 appendectomies: a single-institution study. Int J Colorectal Dis.

[8] Başkent A, Alkan M, Başkent MF (2022). Analysis of appendiceal tumors detected in appendectomy specimens: single center experience. South Clin Ist Euras.

[9] Wang D, Ge H, Lu Y, Gong X (2023). Incidence trends and survival analysis of appendiceal tumors in the United States: primarily changes in appendiceal neuroendocrine tumors. PLoS One.

[10] Chiou YY, Pitman MB, Hahn PF, Kim YH, Rhea JT, Mueller PR (2003). Rare benign and malignant appendiceal lesions: spectrum of computed tomography findings with pathologic correlation. J Comput Assist Tomogr.

[11] Perivoliotis K, Christodoulidis G, Samara AA (2021). Low-grade appendiceal mucinous neoplasm (LAMN) primarily diagnosed as an ovarian mucinous tumor. Case Rep Surg.

[12] Tajima T, Tajiri T, Mukai M (2018). Single-center analysis of appendiceal neoplasms. Oncol Lett.

[13] Skendelas JP, Alemany VS, Au V, Rao D, McNelis J, Kim PK (2021). Appendiceal adenocarcinoma found by surgery for acute appendicitis is associated with older age. BMC Surg.

[14] Ladel L, Tan WY, Jeyakanthan T, Sailo B, Sharma A, Ahuja N (2023). The promise of epigenetics research in the treatment of appendiceal neoplasms. Cells.

[15] Rencuzogullari A, Atar C, Topal U (2023). Analysis of appendiceal neoplasms in 1,423 appendectomy specimens: a 10-year retrospective cohort study from a single institution. Rev Assoc Med Bras (1992).

[16] Yilmaz S, Bolukbasi H (2023). Appendiceal neoplasms: suspected findings and reports of 14 cases. Indian J Cancer.

[17] Kunduz E, Bektasoglu HK, Unver N, Aydogan C, Timocin G, Destek S (2018). Analysis of appendiceal neoplasms on 3544 appendectomy specimens for acute appendicitis: retrospective cohort study of a single institution. Med Sci Monit.

[18] Arellano ML, González-Domínguez Y, Molina-Ortiz F (2016). Primary adenocarcinoma of the appendix: experience at La Paz University Hospital of Madrid (1967-2014). Int J Surg Open.

[19] Chen HT, Lee YT, Wu YK (2006). Primary appendiceal malignancy: a clinicopathologic study. Kaohsiung J Med Sci.

[20] Misdraji J (2015). Mucinous epithelial neoplasms of the appendix and pseudomyxoma peritonei. Mod Pathol.

[21] Barut B, Gönültaş F (2019). Carcinoid tumors of appendix presenting as acute appendicitis. Ulus Travma Acil Cerrahi Derg.

[22] Tchana-Sato V, Detry O, Polus M (2006). Carcinoid tumor of the appendix: a consecutive series from 1237 appendectomies. World J Gastroenterol.

[23] Zhang HW, Jiang Y, Huang ZY, Zhou XC (2023). Analysis of surgical treatment of appendix neuroendocrine neoplasms-17 years of single-center experience. World J Surg Oncol.

[24] Kani V, Ravichandran A, Grace Priyadarshini S, Diwagar N, Esakki M (2024). Multifocal primary extranodal non-Hodgkin lymphoma: a report of a rare case. Cureus.

[25] B N, Grace Priyadarshini S, P J, Chander R V (2024). An unusual case of CD5-positive extranodal marginal zone lymphoma of the mucosa-associated lymphoid tissue (MALT) involving the appendix. Cureus.

[26] García-Norzagaray JC, Villalobos-López JA, Flores-Nájera H, Valle Leal JG, García Torres CD (2019). Primary lymphoma of the appendix: a case report and review of the literature. Rev Gastroenterol Mex (Engl Ed).

[27] Podda M, Gerardi C, Cillara N (2019). Antibiotic treatment and appendectomy for uncomplicated acute appendicitis in adults and children: a systematic review and meta-analysis. Ann Surg.

[28] Davidson GH, Flum DR, Talan DA (2017). Comparison of Outcomes of antibiotic Drugs and Appendectomy (CODA) trial: a protocol for the pragmatic randomised study of appendicitis treatment. BMJ Open.

